# Metabolomics of Type 1 and Type 2 Diabetes

**DOI:** 10.3390/ijms20102467

**Published:** 2019-05-18

**Authors:** Borros Arneth, Rebekka Arneth, Mohamed Shams

**Affiliations:** 1 Institute of Laboratory Medicine and Pathobiochemistry, Molecular Diagnostics, Hospital of the Universities of Giessen and Marburg (UKGM), Justus Liebig University Giessen, Feulgenstr. 12, 35392 Giessen, Germany; 2 Clinics for Internal Medicine 2, University Hospital of the Universities of Giessen and Marburg UKGM, Justus Liebig University. Giessen, 35392 Giessen, Germany; Rebekka.R.Arneth@innere.med.uni-giessen.de; 3 Department of Pharmacy Practice, Faculty of Pharmacy, Mansoura University, Mansoura 35516, Egypt; mshamspharma@gmail.com

**Keywords:** diabetes mellitus type 1, diabetes mellitus type 2, metabolism, biomarkers

## Abstract

Type 1 and type 2 diabetes mellitus (DM) are chronic diseases that affect nearly 425 million people worldwide, leading to poor health outcomes and high health care costs. High-throughput metabolomics screening can provide vital insight into the pathophysiological pathways of DM and help in managing its effects. The primary aim of this study was to contribute to the understanding and management of DM by providing reliable evidence of the relationships between metabolites and type 1 diabetes (T1D) and metabolites and type 2 diabetes (T2D). Information for the study was obtained from the PubMed, MEDLINE, and EMBASE databases, and leads to additional articles that were obtained from the reference lists of the studies examined. The results from the selected studies were used to assess the relationships between diabetes (T1D and/or T2D) and metabolite markers—such as glutamine, glycine, and aromatic amino acids—in patients. Seventy studies were selected from the three databases and from the reference lists in the records retrieved. All studies explored associations between various metabolites and T1D or T2D. This review identified several plasma metabolites associated with T2D prediabetes and/or T1D and/or T2D in humans. The evidence shows that metabolites such as glucose, fructose, amino acids, and lipids are typically altered in individuals with T1D and T2D. These metabolites exhibit significant predictive associations with T2D prediabetes, T1D, and/or T2D. The current review suggests that changes in plasma metabolites can be identified by metabolomic techniques and used to identify and analyze T1D and T2D biomarkers. The results of the metabolomic studies can be used to help create effective interventions for managing these diseases.

## 1. Introduction

Type 1 and/or type 2 diabetes (T1D and/or T2D) are both common chronic diseases, affecting millions of people worldwide, leading to poor health outcomes and increased health care costs [1]. In addition, both diseases (T1D and T2D) are associated with reduced quality of life [2]. The risk factors for T2D include those associated with lifestyle, such as unhealthy eating, as well as genetic factors that interact with each other and an individual’s living environment [2]. Researchers have long attempted to understand T2D to develop interventions and treatment plans that can improve the health and well-being of these patients [2,3,4,5], and it has become increasingly clear over the last decade that high-throughput metabolomic techniques and technologies can provide vital insight into the preconditions (or risk factors) and pathophysiological pathways of both T1D and T2D [6,7,8,9]. Indeed, Guasch-Ferré et al. described metabolomics as the systematic analysis and study of metabolites in a biological sample [1], including low-molecular-weight biochemical compounds such as organic acids, amino acids, sugars, lipids, and nucleotides [3].

Previous studies have analyzed the relationships between various metabolites and T1D and T2D; in most, mass spectrometry together with gas- or liquid-phase chromatography and proton (1H) nuclear magnetic resonance spectroscopy were utilized to assess correlations between metabolites and T2D risk and symptoms [4]. Overall, expansion of the current literature, knowledge of the pathophysiological pathways of T2D and identification of novel and reliable biomarkers can markedly enhance the detection and management of T2D. Moreover, new interventions may be created for caregivers to help and enhance the health and well-being of patients. The current study intends to contribute to the understanding and management of both types of diabetes by providing reliable research evidence of the relationships between metabolites and both types of DM (T1D and T2D). More specifically, this review evaluates available research for associations between specific metabolites, such as amino acids, and T1D and/or T2D, with subsequent analysis of human studies assessing metabolite biomarkers, such as glutamine, glycine, and aromatic amino acids, in T1D and/or T2D patients.

## 2. Methodology

### 2.1. Search and Study Identification

This review entailed a search of electronic databases to identify studies that may help to determine the association between metabolomics and T1D and T2D. The search was performed using the PubMed, MEDLINE, and EMBASE databases. The search terms included “metabolomics,” “metabolite markers,” “type 1 diabetes,” and “type 2 diabetes.” Other outcomes, such as “insulin sensitivity,” “type 1 diabetes,” “type 2 diabetes,” and “insulin sensitivity,” were also used in the search process. Additional sources were obtained from the reference lists of the studies that met the inclusion criteria. All articles, including those obtained from electronic databases and through cross-referencing, were screened via abstract analysis to determine their suitability for inclusion in the review.

### 2.2. Study Selection

The selected studies were assessed based on their titles and abstracts. Only articles that passed the first screening process were considered for full-text review. Two reviewers assessed the studies and selected independent reports based on their quality and suitability for inclusion. Any differences that emerged were addressed through the discussions between the reviewers. Throughout the screening process, the two reviewers were expected to follow clear inclusion and exclusion criteria that had been formulated prior to the study.

Papers were eligible for consideration and inclusion in this review if they described human case-control, case-cohort, or randomized controlled trials or clinical trials. In addition, only studies that examined relationships between specific metabolites and T1D and/or T2D were included. The reviewers excluded animal studies, commentaries, letters, duplicate publications, and editorials from the final lists. Moreover, only studies published in English were considered. It is imperative to note that the search included only publication dates of 2010–2017. The overall goal was to increase the reliability of the search while providing access to a wide range of up-to-date studies related to the topic of interest.

### 2.3. Assessment of Risk of Bias

Before employing the selected studies to investigate the relationships between metabolites and T1D and/or T2D, the risk of bias was assessed, and the internal validity of the studies was evaluated; due to their ability to influence the results, discussion, and conclusions of studies, these measures were critical for the success of this review. Two independent reviewers assessed the risk of bias using predefined assessment criteria. Any discrepancies or challenges that arose during the assessment process were addressed through discussions between the two reviewers. The internal validity of the studies was examined using the risk of bias tool provided by the Systematic Review Center for Laboratory Animal Experimentation (SYRCLE). This process involved examining several factors, including the selection process, performance, and attrition bias, of each study.

### 2.4. Data Extraction and Analysis

A qualitative review of the results and findings of the non-prospective studies included in the final list of the sources was performed, including extracting relevant information and data from the studies, creating a summary of their major findings, and reporting their conclusions in a qualitative manner. For each prospective study, a qualitative review and a quantitative analysis were carried out. During the former, the results of each study were extracted with a specific focus on the relationships between metabolites and T1D and/or T2D. For the latter, associations between specific metabolites and T2D risk were assessed. In addition, this process involved examining quantitative factors and variables, such as measures of uncertainty and multivariable-adjusted effect estimates. No restrictions were applied in terms of heterogeneity; instead, sources of heterogeneity were investigated through careful sensitivity and subgroup analyses. Some of the potential sources of heterogeneity that were considered in the study included the study design, participant status, and condition severity.

## 3. Results and Discussion

### 3.1. Literature Search Results

The researcher worked with two independent reviewers to produce the final list of articles that were used in the current review. A total of 125 records were obtained from the electronic databases accessed and from the reference lists of the studies selected. The abstracts of these studies were reviewed, and those that did not meet the inclusion criteria were removed from the list. Among the 125 abstracts reviewed by the two independent reviewers, 40 did not meet the inclusion criteria and were removed. The remaining 85 studies were subjected to full-text review, which led to the exclusion of 15 sources that were outside the scope of this study and/or failed to meet all the criteria for inclusion.

### 3.2. Study Characteristics

Ultimately, 70 studies were selected from the above databases and from the reference lists of the identified records. The first author and year of publication were used to identify the studies. The main design characteristics included the nature of the metabolites assessed in each study, the participants, and the study design. In particular, the studies examined associations between T1D and/or T2D and low-molecular-weight biochemical compounds, such as organic acids, amino acids, sugars, lipids, and nucleotides. Although each study evaluated different metabolites, the primary focus of each was to demonstrate how metabolites relate to T1D and T2D.

### 3.3. The Insulin Signaling Pathway

Insulin binds to the insulin receptor and activates PI3K and, subsequently, AKT. AKT then inhibits GSK-3β.

As one of the first kinases capable of phosphorylating glycogen synthase (GS), GSK-3β is one of the few protein kinases inactivated by phosphorylation. A high expression of GSK-3β was correlated with a decrease in insulin sensitivity, and GSK-3β was involved in blood glucose regulation, insulin deficiency, and insulin resistance. Because of these results, more and more attention has been paid to studying the relationship between GSK-3β and the occurrence of DM.

Insulin-based regulation is the main path of blood glucose regulation, in which the insulin receptor-mediated signaling pathway plays a key role. 

**Figure 1:**
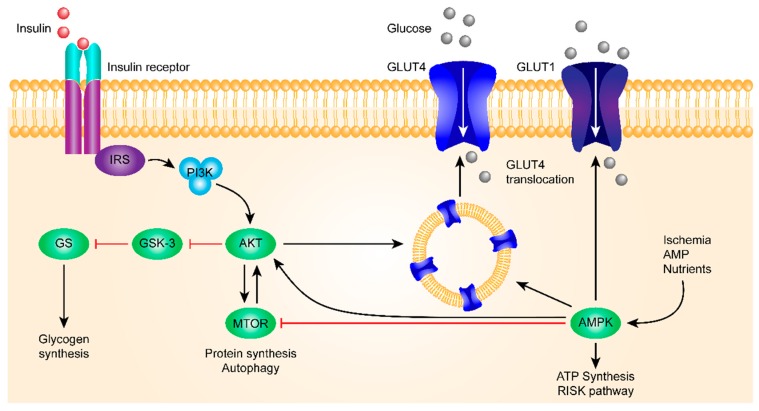
This figure shows the insulin signaling pathway. The insulin receptor binds insulin, has a tyrosine-protein kinase activity, and mediates the metabolic functions of insulin. Binding to insulin stimulates the association of the receptor with downstream mediators, including insulin receptor substrate-1 (IRS-1) and PI3K. The insulin receptor can activate PI3K either directly, by binding to the p85 regulatory subunit, which produces PIP3, or indirectly, which leads to phosphorylation and the activation of AKT. Afterward, AKT phosphorylates the Ser9 site of GSK-3β and inhibits its activity. The PI3K/AKT/GSK-3β signaling pathway is involved in the insulin signaling transduction, and GSK-3β is regulated and controlled by insulin in this signaling pathway, which is related to the glycogen synthesis regulation.

For the insulin signaling pathway, see Figure 1.

Failing to inhibit the activity of GS, which promotes glycogen synthesis and reduces the blood glucose level, results in hyperglycemia and DM. When insulin signal dysfunction occurs, the body increases GSK-3β activity to eventually increase blood glucose, both by phosphorylation of the Tyr216 site of GSK-3β to inhibit the activity of PI3K/AKT and by blocking the signaling pathways to negatively regulate GSK-3β through PI3K/AKT-dependent mechanisms. Studies have shown that heat shock-induced glycogen synthesis in L6 skeletal muscle cells leads to an increase in glycogen-associated protein phosphatase 1 (PP-1) and GS activity, accompanied by sustained AKT/GSK-3β phosphorylation. When Wortmannin (PI3K inhibitor) inhibits AKT/GSK-3β phosphorylation, it prevents 2-deoxyglucose uptake and eliminates heat shock-induced glycogen synthesis. In DM model mice, it was found that the excessive activation of GSK-3β resulted in the decrease of islet β cell proliferation.

There is recent growing evidence that abnormalities in the microbiota composition can play a major role in the development of obesity and diabetes and that some actions of metformin may be mediated by gut bacteria. Several mechanisms have been found, including an association between reduced and altered microbial diversity and inflammation, insulin resistance, and adiposity. In particular, a rise in the Firmicutes/Bacteroidetes ratio is related to low-grade inflammation and to an increased capability of harvesting energy from food. Interestingly, a high-fat diet favors the growth of bacteria capable of extracting more energy from food.

### 3.4. Metabolites and Type 1 Diabetes

A review of the identified studies revealed that several researchers have explored relationships between metabolites and T1D [10,11,12]. T1D is a chronic autoimmune disease that occurs when the body is not able to produce insulin due to the destruction of insulin-producing cells in the pancreas. In other words, T1D is characterized by the inability of the body to produce insulin. Research has shown that certain changes in metabolites appear to correlate with increased T1D risk [13,14,15,16]. In addition, analysis of metabolites facilitates the diagnosis and management of T1D, thus explaining numerous attempts to elucidate relationships between T1D and various metabolites.

One type of metabolite that has been studied with respect to the risk and occurrence of T1D is amino acids. More specifically, previous studies have linked the aromatic amino acids phenylalanine and tyrosine to an increased risk of developing T1D [17,18,19,20,21,22].

Additional studies have reported that branched-chain amino acids promote insulin action and signaling processes, whereas others have demonstrated that they worsen insulin resistance in patients with T1D [23,24,25].

Such conflicting results show that the etiology of T1D and its association with branched-chain amino acids are complex and incompletely understood. One explanation for the association between these amino acids and T1D is their high concentrations in obese individuals [26,27,28,29,30,31,32]. Others have noted that a high intake of valine, leucine, and isoleucine within the context of a high-fat diet can lead to insulin resistance, which can compromise the physiological capacity of insulin to manage and suppress levels of branched-chain amino acids in humans [23,33,34]. Regardless, further investigation is needed if such conclusions are to inform T1D management.

Other studies have associated T1D autoimmune development with lipidomic changes. Specifically, changes in the metabolomic profiles of different classes of lipids, such as plasma phospholipids, triglycerides, cholesterol esters, sphingolipids, and glycerophospholipids, within the context of T1D have been addressed [34,35,36,37,38,39,40]. Levels of low-carbon-number saturated lipid classes, such as palmitic, myristic, and stearic acid, tend to be higher in individuals with T1D than in individuals without diabetes [41,42,43,44,45]. Notably, some researchers have reported reduced concentrations of sphingomyelins and glycerophospholipids in T1D patients [36].

**Figure 2:**
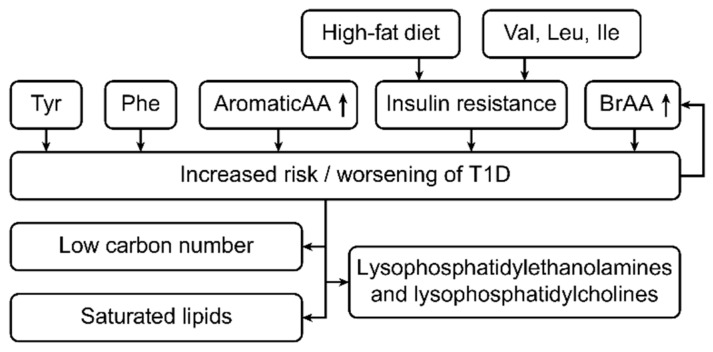
This figure shows the metabolic changes in type 1 diabetes (T1D) patients. The risk for developing type 1 diabetes increases with increases in the concentrations of phenylalanine and branched-chain amino acids (BRAAs). In contrast, if a patient already has type 1 diabetes, a high BRAA concentration worsens the disease state [23,33,34]. Additionally, in type 1 diabetes, a high-fat diet and high levels of the amino acids Val, Leu and Ile increase the levels of insulin resistance [23,33,34]. In addition, type 1 diabetes is associated with increased levels of low-carbon-number saturated lipids, lysophosphatidylethanolamine and lysophosphatidylcholine [39].

In another study, lysophosphatidylethanolamine and lysophosphatidylcholine contents were reported to be higher in those with T1D than in those without diabetes [39]. Overall, these associations parallel the conventional understanding of the metabolic basis of T1D and insulin resistance. Figure 2 shows the metabolic changes in type 1 diabetes patients.

### 3.5. Metabolites and Type 2 Diabetes

T2D is a nutritional disorder characterized by the inability of the body to respond to insulin and is associated with many complications, including kidney failure, retinopathy, lower-limb amputation, and an increased risk of cardiovascular disease [1,46] and stroke [47]. Although researchers have associated several risk factors with T2D [46], the actual extent to which each of these factors leads to the development of T2D remains unclear. Some commonly cited risk factors include obesity, high cholesterol and blood sugar levels, family history of T2D, and history of gestational diabetes [48,49,50].

The concept of metabolomics has also been applied in the study and understanding of T2D. A review of selected studies revealed a growing body of research demonstrating relationships between T2D and metabolomic profiles [46], and researchers have examined how the existence and level of metabolites, such as amino acids, can facilitate understanding, diagnosing, and predicting the occurrence of T2D [48,49,50]. In general, these studies have compared the concentrations of metabolites in individuals with diabetes to those without the disease [50,51]. Other reports have focused on associations between metabolites and prediabetes measures, such as glucose tolerance [1,52,53].

In patients with T2D, the concentrations of branched-chain amino acids can be up to 1.5-fold or even 2-fold higher than those in healthy subjects, with a mean change below 1.5-fold (1.2 to 1.3). These results are reproducible and statistically significant [1,3,23].

McCormack et al. explored the relationship between branched-chain amino acids and T2D, focusing on the total concentration of all three branched-chain amino acids, i.e., leucine, isoleucine, and valine, in overweight children. A study population of 69 subjects was enrolled in their cross-sectional cohort study, with the researchers determining high concentrations of branched-chain amino acids to be associated with insulin resistance among the study subjects [23]. Based on the results, the researchers concluded that an increased concentration of branched-chain amino acids is a reliable predictor of future insulin resistance among T2D patients.

Significant associations between T2D and amino acids can help in managing the disorder [54,55,56,57,58,59,60]. For instance, a study by Newgard reported higher plasma levels of branched-chain and aromatic amino acids and higher glutamate-to-glutamine ratios in patients with diabetes than in healthy individuals. These findings are in accordance with those of studies reporting a positive association between prediabetes in obese individuals and elevated branched-chain amino acids and glutamate-to-glutamine ratios.

In contrast, Menni et al. employed an untargeted metabolomic approach to explore links between T2D and branched-chain amino acids and their derivatives in a study that involved 2204 women from the United Kingdom [61], with branched-chain amino acids being associated with impaired fasting glucose levels in patients with T2D. In other studies, amino acids (such as hydroxyl acids and hydroxybutyrate) were found to lead to elevated insulin resistance and impaired glucose tolerance in T2D patients [62,63,64,65,66,67,68]. In addition, β-hydroxybutyrate and 3-hydroxybutyrate have been correlated with a higher risk of prediabetes.

Unlike for T1D, only a small number of studies have focused on relationships between the metabolomic lipid profiles and T2D, as examined through high-throughput techniques to identify various classes of lipids, such as plasma phospholipids, triglycerides, sphingolipids, and glycerophospholipids [69]. Indeed, high concentrations of low-carbon lipids, such as glycerophospholipids and sphingomyelins, have been found in individuals suffering from T2D. As some studies have linked T2D to increased levels of fatty acids, such as dodecanoic and myristic acids [70], the identification and profiling of these lipids may facilitate the prediction and management of T2D [1,70].

Sugar metabolites and organic acids have also been profiled and studied to promote our understanding and management of T2D. For example, a small number of studies have reported positive associations with T2D for sugar metabolites, such as glucose, dihexose, glucose, mannose, arabinose, and fructose.

**Figure 3:**
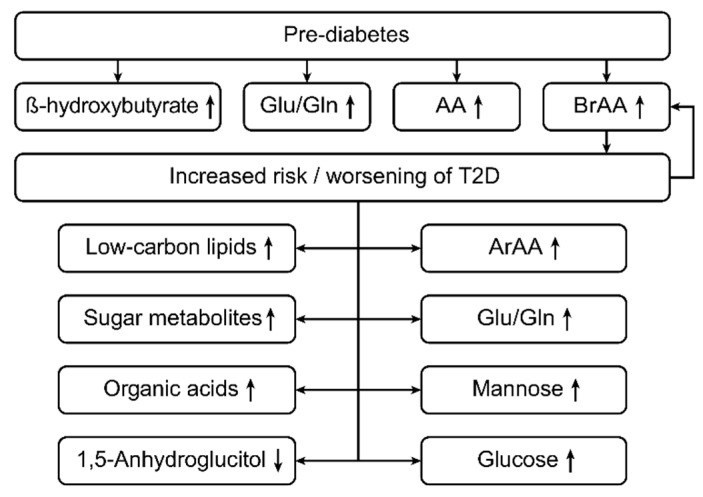
This figure shows the metabolic changes in type 2 diabetes (T2D) patients. In type 2 diabetes, the levels of branched-chain amino acids (BRAAs), aromatic amino acids (ArAAs) and the glutamine/glutamate ratio (Glu/Gln) are increased [1,3,23,25]. Prior to the onset of diabetes mellitus (DM) type 2, the levels of BRAAs, ArAAs, and ß-hydroxybutyrate and the Glu/Gln ratio are increased, even in prediabetic patients [25]. In type 2 diabetes, the levels of low-carbon lipids, sugar metabolites, organic acids, mannose and glucose are increased, but the levels of 1,5-anhydroglucitol are decreased [71].

A case-control study performed by Suhre showed that plasma levels of 1,5-anhydroglucitol were approximately 37.8 percent lower in people with T2D than in healthy individuals [71] and that levels of glucose, desoxyhexose, mannose, and dihexose were higher in subjects with diabetes than in controls [71]. In addition, certain organic acids, such as acetic acid, dimethyl ester, and maleic acid, have been associated with T2D, and a few isolated studies have linked other organic compounds, such as purines and urea cycle metabolites, including arginine, citrulline, and ornithine, to T2D [54]. Nonetheless, further investigations are needed to support the findings of such limited studies. Figure 3 shows the metabolic changes in type 2 diabetes patients.

### 3.6. Pathobiochemical Considerations

According to the recent studies described above, patients with T1D and T2D exhibit elevated levels of aromatic and branched-chain amino acids [34,56,57,58]. The aromatic amino acids phenylalanine, tyrosine, and tryptophan, as well as the branched-chain amino acids valine, isoleucine, and leucine, are essential amino acids in humans; that is, these amino acids must be obtained from food. The main producers of these amino acids are intestinal bacteria, especially *Escherichia coli* [11,32,72].

Erythrose 4-phosphate, the starting material for the shikimic acid metabolic pathway for generating aromatic amino acids in intestinal bacteria such as *E. coli* [11,32,72], appears to play a role in the relationship between elevated levels of aromatic amino acids and type 2 diabetes mellitus. In addition, erythrose 4-phosphate is an intermediate of the pentose phosphate pathway, a central metabolic pathway for the utilization of glucose in humans, intestinal bacteria, and most living organisms [72].

Entry of a massive oversupply of glucose into the pentose phosphate pathway in human cells, as well as in intestinal bacteria, can be assumed to result in elevated levels of erythrose 4-phosphate. In intestinal bacteria, erythrose-4-phosphate then enters the shikimic acid pathway, which appears to lead to increased formation of aromatic amino acids [72].

**Figure 4:**
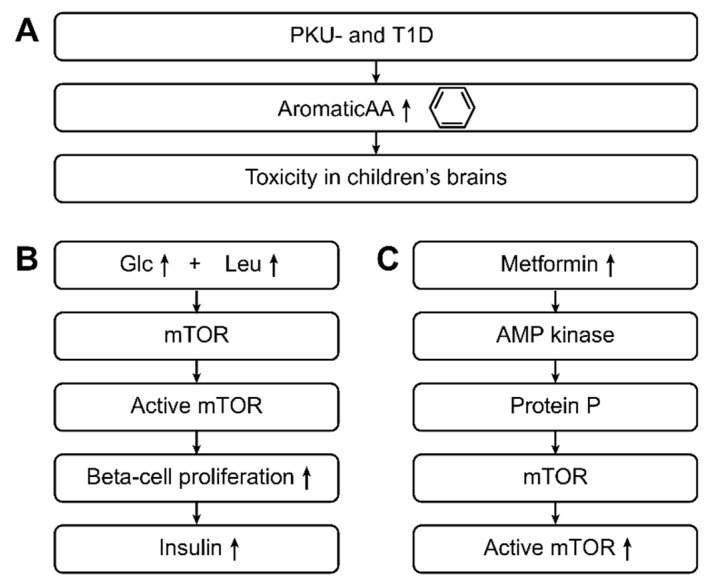
This figure shows the pathophysiological aspects associated with the metabolic changes in type 1 and type 2 diabetic patients. Aromatic amino acids (ArAAs) are toxic to children’s brains, and this toxicity can be observed in PKU- and T1D-positive children [72]. An important pathophysiological pathway is the following: High glucose and high leucine levels lead to the activation of rapamycin (mTOR), and activated mTOR leads to beta cell proliferation and a greater release of insulin [72]. The previous pathway can also be induced by metformin. Specifically, through AMP-kinase and subsequent protein-P activation, metformin leads to mTOR activation, leading to increased insulin levels. Through this pathway, metformin acts as a mild drug in type 2 diabetes.

The pathway involving branched-amino acids (essential amino acids for humans) is similar and begins with pyruvic acid, the production of which is increased when high glucose levels are available. Furthermore, in the presence of high glucose levels, even more pyruvic acid is available to enteric bacteria, and more pyruvic acid is subsequently produced. Together with pyruvic acid, high amounts of the resulting amino acids valine, leucine, and isoleucine (branched-chain amino acids) are produced. Figure 4 gives the pathophysiological aspects associated with metabolic changes.

### 3.7. Cell Signaling: The Role of Branched-chain Amino Acids (valine, leucine, and isoleucine) in T1D and/or T2D

With respect to cell signaling, the mechanistic target of the rapamycin (mTOR) pathway has an important role in beta-cell growth and subsequent insulin secretion. High concentrations of glucose in the blood activate mTOR signaling, with leucine playing an indirect role.

Overall, the combination of glucose, leucine, and other activators stimulate the mTOR pathway, inducing the proliferation of beta-cells and insulin secretion. High concentrations of leucine cause mTOR pathway hyperactivity, resulting in activation of S6 kinase and leading to inhibition of insulin receptor substrates through serine phosphorylation. In cells, increased activity of the mTOR complex eventually causes an inability of beta-cells to release insulin through an inhibitory effect on S6 kinase, which leads to cellular insulin resistance and contributes to the development of T2D.

The occurrence of branched-chain amino acid signatures that lead to insulin resistance has been studied in both humans and rats. In humans, the body mass indices of subjects have been compared to the concentrations of branched-chain amino acids in their diets as well as their insulin resistance levels. Subjects who are considered obese have higher metabolic signatures of branched-chain amino acids and higher resistance to insulin than do lean individuals with a lower body mass index. In addition, rats fed a diet rich in branched-chain amino acids display increased rates of insulin resistance and impaired phosphorylation of enzymes within their muscles. In contrast, obese mice with pre-diabetes fed a low-branched-chain amino acid, calorie-unrestricted, high-fat, and high-sugar diet experienced an improvement in metabolic health, whereby an unhealthy but low-branched-chain amino acid diet promoted leanness and increased blood sugar regulation [73].

**Figure 5:**
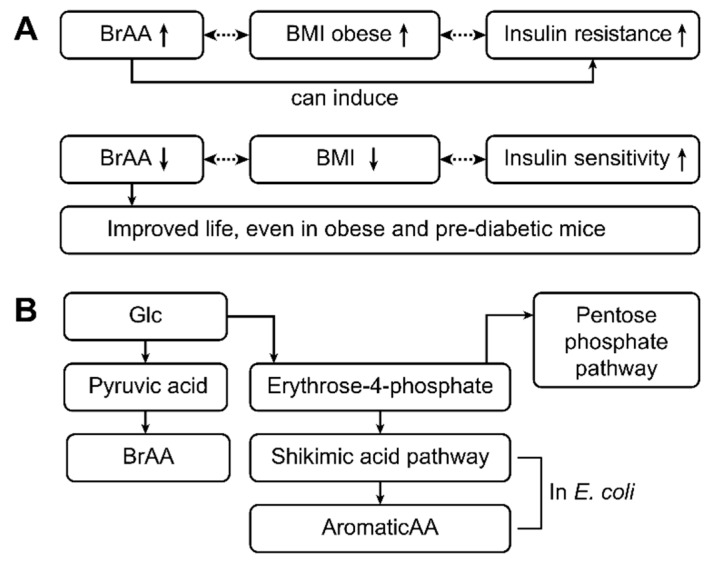
This figure shows the pathophysiology and pathways related to the metabolic changes that occur in patients with type 1 and type 2 diabetes. High levels of branched-chain amino acids (BRAAs) are often associated with increased BMI levels (obesity) and insulin resistance (type 2 diabetes) [23,24,25]. In contrast, low levels of BRAAs are accompanied by lower BMIs and good insulin sensitivity. Thus, BRAAs appear to induce insulin resistance. Decreasing the BRAA concentration might improve life, as has been shown in obese and prediabetic mice [73]. BRAAs are synthesized from glucose via pyruvic acid. Glucose is normally used in the pentose phosphate pathway, and erythrose-4-phosphate is obtained as an intermediate product. In contrast, erythrose-4-phosphate is an important educt of the shikimic acid pathway, which is an important pathway in enteral *E. coli*. As a result, aromatic amino acids are produced by *E. coli* from erythrose-4-phosphate [11,32,72] and from glucose in patients with type 2 diabetes mellitus and high glucose concentrations [72].

Metformin can activate the adenosine monophosphate kinase (AMP kinase), which phosphorylates proteins involved in the mTOR pathway and leads to progression of the mTOR complex from its inactive state to its active state. Metformin has been suggested to act as a competitive inhibitor of the amino acid leucine in the mTOR pathway [74]. Figure 5 gives further pathophysiological aspects associated with metabolic changes in type 1 and type 2 diabetes.

### 3.8. What Came first the Chicken or the Egg? 

To date, it is unclear whether the observed metabolic changes are a consequence of high glucose levels and therefore of the diseases T1D and/or T2D or if the metabolic changes are causative and lead to the development of T1D and/or T2D. We only observed the co-occurrence of both events: Diabetes and metabolic changes. It seems plausible that both events occur “hand in hand”, especially in T2D; thus, metabolic changes occur before or with the development of T2D.

### 3.9. Clinical Impact

To date, metabolic changes other than the high glucose levels and glycosylated hemoglobin are not used as targets in patient treatment. Further studies are needed to determine if the other metabolic changes described here can be used for improved treatment of patients with T1D and/or T2D.

### 3.10. Effects of Polyphenols and Resveratrol on Metabolism-Patterns in Patients and Mice with Type 1 Diabetes 

Polyphenols and resveratrol are components of grapes and other pharmacologically relevant plants (so called phytopharmica).

Studies have shown that polyphenols are beneficial for a dietary modulation of the gut-brain-microbiome axis, which is able to alter both glucose regulation and insulin resistance in prediabetic and diabetic patients [75]. Metabolomic alterations in diabetes patients have also been influenced positively by polyphenols’ ability to correct the patient’s metabolic pattern. 

Studies of diabetes and obesity in animal models have shown that resveratrol mitigates complications from diabetes-induced metabolic diseases, beyond those resulting from oxidative stress. Results obtained with cultured preadipocytes have revealed that prolonged resveratrol treatment impairs adipogenesis. Further, oral administration of resveratrol has been demonstrated to improve glucose homeostasis in obese individuals [76]. 

These studies all seem to indicate connections between the gut-brain-microbiome axis and changes in metabolic patterns. For example, taxonomic and predicted functional changes in the gut microbiome of obese mice were normalized by resveratrol ingestion. In particular, resveratrol-induced changes in the gut microbiome were characterized by a decreased relative abundance of Turicibacteraceae, Moryella, Lachnospiraceae, and Akkermansia and an increased relative abundance of Bacteroides and Parabacteroides. Fecal transplantation from healthy resveratrol-fed donor mice is sufficient to improve glucose homeostasis in obese mice, and this suggests that resveratrol-mediated changes in the gut microbiome may play an important role in the mechanism behind the changes seen in patients using resveratrol [76]. 

## 4. Conclusions

The purpose of this review was to explore current research data concerning the relationships between metabolites and T1D and T2D. By analyzing research evidence from previous studies addressing this subject, this review identified several plasma metabolites associated with prediabetes, T1D, and T2D in humans. These metabolites were usually identified through high-throughput metabolomic techniques.

The available body of research evidence shows that metabolites, such as glucose, fructose, amino acids, and lipids, are typically altered in individuals with T1D and T2D. In addition, these metabolites show significant prospective and predictive associations with prediabetes, T1D, and T2D. The results of the current review suggest that changes in plasma metabolites can be identified through metabolomic techniques, which can also be utilized to identify T1D and T2D biomarkers. Because the management of all forms of diabetes includes dietary and lifestyle interventions, future studies focusing on elucidating metabolites that can modulate the impacts of dietary intake are needed, as are investigations regarding how diet impacts metabolites in the body.
